# Exploratory study comparing a single episode of feedback with regular feedback and no feedback on BVM ventilation during a simulated cardiac arrest over a six-month time frame: a research protocol

**DOI:** 10.29045/14784726.2025.9.10.2.34

**Published:** 2025-09-01

**Authors:** Graham McClelland, Owen Finney, Karl Charlton, Benjamin Kirk, Laura Blair, Sarah Hepburn

**Affiliations:** Northumbria University ORCID iD: https://orcid.org/0000-0002-4502-5821; North East Ambulance Service NHS Foundation Trust ORCID iD: https://orcid.org/0000-0003-3744-2710; North East Ambulance Service NHS Foundation Trust ORCID iD: https://orcid.org/0000-0003-3744-2710; North East Ambulance Service NHS Foundation Trust ORCID iD: https://orcid.org/0000-0003-3744-2710; North East Ambulance Service NHS Foundation Trust ORCID iD: https://orcid.org/0000-0003-3744-2710; North East Ambulance Service NHS Foundation Trust ORCID iD: https://orcid.org/0000-0003-3744-2710

**Keywords:** out-of-hospital cardiac arrest, skill maintenance, ventilation

## Abstract

**Introduction:**

Out-of-hospital cardiac arrest (OHCA) remains a major cause of mortality in the UK, with survival rates remaining low despite advancements in resuscitation techniques. The European Resuscitation Council and Resuscitation Council UK guidelines recommend controlled ventilation during cardiopulmonary resuscitation (CPR), yet studies show that ambulance clinicians often fail to meet these standards. In particular, hyperventilation has been linked to worse outcomes. This protocol describes a study that will explore the impact of different applications of real-time ventilation feedback on the quality of ventilations during CPR in a simulated environment.

**Methods:**

This exploratory simulation study will assess the effectiveness of real-time feedback on the quality of ventilations delivered by ambulance clinicians. Participants from North East NHS Foundation Trust will be randomly assigned to three arms, receiving a single episode of feedback, regular feedback or no feedback (used as a control group). Each arm will complete four simulated OHCA scenarios over six months, and their ventilation quality will be assessed at each session. The primary outcome will be the quality of ventilations, measured by rate and tidal volume, at the six-month mark. Secondary outcomes include trends in ventilation quality over time and participant characteristics.

**Discussion:**

This study aims to explore whether regular feedback improves the quality of ventilations during CPR and whether feedback sessions influence skill retention over a six-month period. Findings could inform training strategies, highlighting the role of real-time feedback in maintaining high-quality CPR skills. With a lack of prior research on ventilation skill maintenance in the UK, this study is expected to provide valuable insights into optimising clinical performance.

## Introduction

Out-of-hospital cardiac arrest (OHCA) is the sudden cessation of effective circulation due to absence of cardiac pump function in an out-of-hospital setting (Myat et al., 2018). In the UK in 2023, National Health Service (NHS) ambulance services treated approximately 29,625 OHCAs, with a low survival rate of 9% (Warwick University OHCAO Project, 2023). When treating an OHCA, ambulance clinicians are required to deliver chest compressions and manual ventilations as part of cardiopulmonary resuscitation (CPR). Adequate ventilation is an important aspect of CPR (Olasveengen et al., 2017). Hyperventilation is a critical issue that has received little attention, despite its association with increased intrathoracic pressure and impaired haemodynamics and cardiac output, as well as cerebral vasoconstriction, which can significantly reduce brain perfusion (Kirk et al., 2023). Ultimately, hyperventilation during CPR can be deleterious to survival (Aufderheide & Lurie, 2004; Pitts & Kellermann, 2004).

The European Resuscitation Council (ERC) adult advanced life support (ALS) guidelines recommend ventilating cardiac arrest patients at a rate of 8–12 ventilations per minute with a tidal volume sufficient to produce chest rise (Soar et al., 2021). A previous version of these guidelines specified a tidal volume of 500–600 ml and warned against hyperventilation (Soar et al., 2015). These recommendations are largely mirrored by the Resuscitation Council UK guidelines (Resuscitation Council UK, 2021) and UK ambulance service guidelines (JRCALC, 2023). Despite this, research suggests that 80% of caregivers hyperventilate cardiac arrest patients (Nehme & Boyle, 2009). Previous research has shown that compliance with ventilation guidelines can be improved by the use of feedback (Khoury et al., 2019), in the same way that real-time feedback has improved the quality of compressions. There is currently a lack of data surrounding the use of real-time ventilation feedback devices in OHCA and until very recently it has not been possible to measure the quality of ventilations (Drennan et al., 2024).

A recent manikin study by Charlton et al. (2021) identified that ambulance clinicians delivered poor-quality ventilations, such as hyperventilation or hypoventilation, in respect to both the tidal volume and ventilation rate. The Charlton et al. study showed that many clinicians did not meet ERC guidelines without feedback, but that when feedback was provided, compliance with ERC guidelines significantly improved.

Ventilation with a bag-valve-mask (BVM) is a skill used by ambulance clinicians globally. The JRCALC advanced life support guidelines states: ‘*The initial approach to airway management should usually be BVM*’ (JRCALC, 2023), which aligns with Resuscitation Council ALS guidelines that state: ‘*During CPR, start with basic airway techniques and progress stepwise according to the skills of the rescuer until effective ventilation is achieved*’ (Resuscitation Council UK, 2021). The American Association of EMS Physicians (NAEMSP) recently published a position statement stating that BVM is: ‘*a fundamental airway management skill for all Emergency Medical Services (EMS) clinicians*’ (Lyng et al., 2022). However, the American Heart Association cautions: ‘*BVM is a challenging skill that requires considerable practice for competency*’ (Kleinman et al., 2015).

Outside of clinical practice, ventilation competency, including use of the BVM, is delivered through infrequent training sessions. Unless an individual seeks out relevant activities or completes refresher training provided by their organisation, which is often delivered on an infrequent basis, their skills are likely to decay (Woodman et al., 2021). An initial literature search failed to identify any literature on the maintenance of BVM skills by UK paramedics, although there is literature on other airway skills and maintenance from other settings, such as completing high-fidelity simulation to maintain endotracheal intubation competency (Pilbery, 2018; Youngquist et al., 2008).

If we accept that BVM ventilation is a core ambulance clinician skill, that competence fades over time and that clinicians receive little refresher training or feedback, then it is unsurprising that studies have shown that clinicians struggle to ventilate with a BVM according to guidelines (Charlton et al., 2021; Khoury et al., 2016). However, studies have also shown that real-time feedback can improve the ability of clinicians to ventilate according to guidelines (Charlton et al., 2021; Khoury et al., 2016), but what the impact of occasional or regular access to feedback would be remains unclear.

The NHS ambulance services resuscitate around 30,000 patients with cardiac arrest annually (Perkins et al., 2015; Warwick Clinical Trials Unit, 2021), and a BVM will be used in most of these cases. A BVM will also be used in treatment of other conditions, such as respiratory arrest, so the number of cases where a BVM is used will be higher than 30,000. Therefore, exploring what impact occasional feedback, regular feedback and the current standard of no feedback has on BVM skill maintenance is potentially very important.

### Aims and objectives

The aim of this project is to explore the impact of different applications of real-time ventilation feedback on ambulance clinicians’ ability to deliver high-quality ventilations during CPR in a simulated setting and to explore if skills change over a six-month period.

**Primary objective:** The primary objective of this project is to explore the impact of a single session of feedback compared with regular feedback and no feedback on ventilation quality.

**Secondary objective:** The secondary objective of this project is to explore whether ventilation skills change over a six-month period with repeated assessment.

## Methods

### Trial design and setting

The study will use a randomised experimental design. This study is exploratory in nature and will use a simulated OHCA scenario and volunteer clinicians from a single NHS ambulance service. Participants will be randomised 1:1:1 to three arms:

- Ventilation feedback at the first session only (single feedback)
- Ventilation feedback at all sessions (regular feedback)
- No feedback (control arm).

This study will be conducted by arranging study sessions at sites across the North East NHS ambulance service and inviting participants to complete the scenario at the appropriate time (months 0, 2, 4 and 6) (Table 1). Multiple dates will be arranged to facilitate participation.

**Table 1.** Summary of the study structure.

	Session 1 (month 0)	Session 2 (month 2)	Session 3 (month 4)	Session 4 (month 6)
**Regular feedback**	Feedback	Feedback	Feedback	Feedback
**Single feedback**	Feedback	No feedback	No feedback	No feedback
**Control**	No feedback	No feedback	No feedback	No feedback

The scenarios will be delivered in a standardised manner, using the same equipment each time. Each scenario will last approximately six minutes, which equates to approximately three two-minute cycles of CPR. Where it is not possible to include pairs of participants, a member of the research team may have to perform the chest compressions. Each study session should last less than one hour per participant, which will include a briefing session, the actual simulation and a debrief period with the participants.

### Randomisation procedure

A pre-generated randomisation will be used, with participants allocated sequentially when they attend session 1 at month 0.

### Eligibility criteria

Any operational ambulance staff (paramedics and non-paramedics) employed by North East NHS ambulance service who use a BVM to ventilate as part of their role will be eligible to participate.

### Sample size

This is an exploratory study, so there is no formal sample size or power calculation. The study will use a mix of sampling methods, with one group being a non-probability convenience sampling and a sub-group being sampled via purposive sampling.

The study will aim to recruit between 18 and 30 participants from North East NHS ambulance service operational ambulance staff. This target was selected for pragmatic reasons and the resources available for the study. If more than 30 participants volunteer, it will be the first 30 staff who are included. Up to 40 North East NHS ambulance service Hazardous Area Response Team (HART) paramedics will be offered the chance to participate in the study, but will be analysed as a sub-group in addition to the main analysis. The HART group will be analysed as a separate sub-group due to the participants having different baseline characteristics, such as their training.

### Participant recruitment

Participants will be recruited via North East NHS ambulance service internal media, social media and personal contacts. Participants will be asked to complete a consent form prior to study participation after they have had sufficient time (>24 hours) to consider the participant information. Consent will be checked at the start of each session.

Participants who complete all four study sessions will receive a CPD certificate and a £25 gift voucher in appreciation for their time and input. Participants will also be offered the chance to receive individualised feedback based on the study data and a copy of the final study results.

### Interventions

**Figure 1:**
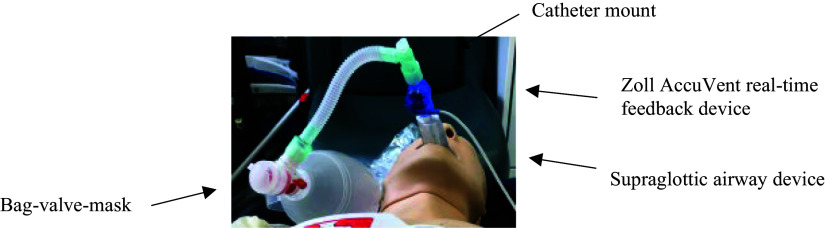
Figure 1. Zoll AccuVent real-time feedback device.

The Zoll AccuVent real-time feedback device is an adjunct placed between the supraglottic airway device or endotracheal tube (ETT) and the catheter mount (all standard pieces of equipment used by North East NHS ambulance service clinicians when managing an airway), which is attached to the Zoll defibrillator as in Figure 1. The Zoll AccuVent provides visual feedback, displaying the rate and tidal volume of each ventilation in the form of a pictorial guide of a circle that fills during each ventilation, and has the capacity to record and download data following each use.

**Figure 2:**
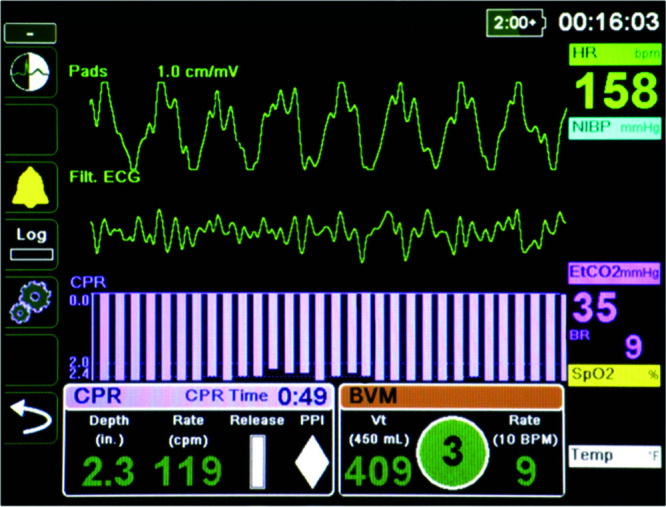
Figure 2. Zoll AccuVent visual feedback.

The visual displays are colour coded to ease interpretation (Figure 2). The Zoll AccuVent real-time feedback device is similar to the chest compression feedback provided by the Zoll defibrillators already used in the North East NHS ambulance service and which is familiar to clinicians.

### Outcomes

The primary outcome for this study will be the quality of the ventilations measured by a combination of rate and volume at month 6 compared between the three arms of the study. Primary outcome data will be tested using either a one-way ANOVA or Kruskal-Wallis test, depending on the distribution (normality) of the data.

Descriptive data will also be reported on secondary outcomes, including:

- trends in ventilation quality
- participants factors, including: age; sex; job role; any recent CPR/ventilation training received; prior involvement in the VANZ1 or VANZ2 studies (previous studies that form the knowledge base for this study conducted by the study team (Charlton et al., 2021)); and recent experiences of delivering ventilations in practice.

As scenarios may be repeated with pairs of participants, we will also look at whether there is a difference between people who provide ventilations first or second.

### Data collection

Source data for the study will consist of data downloaded from the Zoll defibrillator and participant-reported data collected at each session. Source data will stay within the North East NHS ambulance service. Only anonymised data will leave the North East NHS ambulance service and will be shared with Northumbria University for analysis.

### Missing data

In the event that ventilation data cannot be retrieved from the defibrillator then that participant/scenario will be removed from the study. If participants fail to complete all four sessions, their data will still be included for the sessions they completed.

### Data analysis

Data will be collected in Excel, and SPSS will be used for statistical analysis. Primary outcome data will be tested using either a one-way ANOVA or Kruskal-Wallis test depending on the distribution (normality) of the data. Descriptive data will also be reported on secondary outcomes. The HART participants will be analysed as a sub-group using the same statistical analysis due to the participants having a different skill set and cardiac arrest exposure compared to the rest of the participants.

### Ethics

This study will be conducted in accordance with the principles of the Declaration of Helsinki and Good Clinical Practice guidelines. Health Research Authority approval has been granted (Reference number: 24/HRA/4702). Ethical approval was granted by Northumbria University (Reference number: 7882). NHS Research Ethics Committee approval is not required for this study, as study participants are NHS staff and not patients in a clinical setting.

### Dissemination

The results of the study will be submitted for publication in the *British Paramedic Journal* and submitted to relevant conferences, such as the College of Paramedics National Conference and the 999 EMS Research Forum. A summary report will be presented to the North East NHS ambulance service and Northumbria University. Finally, the ubiquity of social media will be utilised to highlight outputs from the study.

## Author contributions

The protocol was developed by GM with support from OF, KC, BK, LB and SH. GM acts as the guarantor for this article.

## Conflict of interest

GM is on the editorial board for the BPJ.

## Funding

Funding from the College of Paramedics Small Research Grant and the North East NHS ambulance service has been used to support this research.
